# C-856-06. Pain and Pruritus Increase Long-term Psychosocial Morbidity in Pediatric Burn Survivors

**DOI:** 10.1093/jbcr/irag033.098

**Published:** 2026-04-06

**Authors:** Anika Y Kim, Bill Insko, Kara McMullen, Sarah A Stoycos, Karen J Kowalske, Colleen M Ryan, Jeffrey C Schneider, Gretchen J Carrougher, Haig A Yenikomshian

**Affiliations:** Division of Plastic and Reconstructive Surgery, Keck School of Medicine, University of Southern California, Los Angeles, CA; Department of Rehabilitation Medicine, University of Washington, Seattle, WA; Department of Rehabilitation Medicine, University of Washington, Seattle, WA; Keck School of Medicine, University of Southern California, Los Angeles, CA; Department of Physical Medicine and Rehabilitation, University of Texas Southwest Medical Center, Dallas, TX; Massachusetts General Hospital, Harvard Medical School, Boston, MA; Spaulding Rehabilitation Hospital, Mass General Brigham, Harvard Medical School, Boston, MA; University of Washington Medicine Regional Burn Center, Seattle, WA; Division of Plastic and Reconstructive Surgery, Keck School of Medicine, University of Southern California, Los Angeles, CA

## Abstract

**Introduction:**

While pain and pruritus are well-established sequelae of burn injury, their longitudinal impact on functional and psychosocial symptoms of pediatric burn survivors is poorly characterized. While prior studies have focused on the acute recovery phase, less is known about how these common and distressing sequelae affect longer-term recovery. This project addresses this gap by evaluating the associations of pain and pruritus with functional and psychosocial symptoms at 12 months post-injury in pediatric burn survivors.

**Methods:**

This retrospective review utilized a multicenter longitudinal database (injured between the years of 2014 and 2024) to identify pediatric burn survivors aged 8–17 years who completed a 12-month after injury self-report survey assessing pain and pruritus. PROMIS domains included pain intensity, pruritus, physical function, anxiety, depression, and fatigue. General linear models (GLM) were fitted at the 12-month follow-up to evaluate independent associations between pain and pruritus scores and demographic (age, sex, race/ethnicity, TBSA burned) and psychosocial variables. Separate models were also constructed to assess the impact of pain and pruritus on physical function, anxiety, depression, and fatigue at the 12-month follow-up.

**Results:**

The study included 67 pediatric burn survivors with a mean age of 11.8, 70.2% male, a mean TBSA of 32.2% (SD 21.3), and 50.8% Hispanic ethnicity (see Table 1). In multivariate regression models at 12 months post-injury, demographic and burn characteristics (age, sex, race, and TBSA) were not significant predictors of itch or pain. However, higher anxiety scores were an independent predictor of greater itch severity (*p*=.0005). No demographic or clinical characteristics independently predicted pain severity. Higher itch was significantly associated with worse physical function (*p*=.0396), anxiety (*p*<.0001), depression (*p*=.0006), and fatigue (*p*=.0096). Higher pain was associated with greater anxiety (*p*=.0109), depression (*p*=.0021), and fatigue (*p*=.0053), but not with physical function (*p*=.1457).

**Conclusions:**

This ten-year review shows that even one year after injury, pain and pruritus remain strongly associated with psychosocial distress, with pruritus additionally linked to impaired physical function. These findings highlight that pain and itch are interrelated drivers of impaired quality of life in children. For pediatric patients, these consequences are particularly detrimental, as they occur during critical years of growth, education, and identity formation.

**Applicability of Research to Practice:**

These results highlight the importance of early recognition and intervention by providers to not only mitigate the downstream effects of pain and pruritus after burn injury but also to promote optimal reintegration and quality of life for pediatric burn survivors.

**Funding for the study:**

The contents of this abstract were developed under a grant from the National Institute on Disability, Independent Living, and Rehabilitation Research (NIDILRR grant number 90DPBU0007). NIDILRR is a Center within the Administration for Community Living (ACL), Department of health and Human Services (HHS). The contents of this abstract do not necessarily represent the policy of NIDILRR, ACL, or HHS, and you should not assume endorsement by the Federal Government.

